# A Randomized, Placebo-Controlled Study on the Safety and Efficacy of Daily Ingestion of Green Tea (*Camellia sinensis* L.) cv. “Yabukita” and “Sunrouge” on Eyestrain and Blood Pressure in Healthy Adults

**DOI:** 10.3390/nu10050569

**Published:** 2018-05-06

**Authors:** Mari Maeda-Yamamoto, Mie Nishimura, Nobuyoshi Kitaichi, Atsushi Nesumi, Manami Monobe, Sachiko Nomura, Yukihiro Horie, Hirofumi Tachibana, Jun Nishihira

**Affiliations:** 1 Agri-Food Business Innovation Center, National Agriculture and Food Research Organization (NARO), Tsukuba, Ibaraki 305-8517, Japan; 2 Department of Medical Management and Informatics, Hokkaido Information University, Ebetsu, Hokkaido 069-8585, Japan; mnishimura@do-johodai.ac.jp (M.N.); nishihira@do-johodai.ac.jp (J.N.); 3 Department of Ophthalmology, Health Sciences University of Hokkaido, Ishikari-gun, Hokkaido 002-8072, Japan; nobukita@hoku-iryo-u.ac.jp (N.K.); y-horie@crux.ocn.ne.jp (Y.H.); 4 Institute of Fruit and Tea Science, NARO, Makurazaki, Kagoshima 898-0087, Japan; nesuco@affrc.go.jp; 5 Institute of Fruit and Tea Science, NARO, Shimada, Shizuoka 428-8501, Japan; monobe@affrc.go.jp (M.M.); nonnon7@affrc.go.jp (S.N.); 6 Division of Applied Biological Chemistry, Department of Bioscience and Biotechnology, Faculty of Agriculture, Kyushu University, Fukuoka, Fukuoka 812-8581, Japan; tatibana@agr.kyushu-u.ac.jp

**Keywords:** green tea cv. “Sunrouge” or “Yabukita”, anthocyanins, flavonols, eyestrain reducing effect, blood pressure elevating effect, adiponectin level increasing effect

## Abstract

The green tea (*Camellia sinensis* L.) cultivar “Sunrouge” contains anthocyanins, catechins and flavonols. To determine whether ingesting green tea containing anthocyanins improves visual function and blood pressure (BP) in healthy adults, a randomized, double-blind, placebo-controlled study was performed. A total of 120 healthy subjects, aged between 20 and 60 years and with a systolic BP (SBP) value of ≤125 and <155 and a diastolic BP (DBP) value <95, or a DBP of ≤75 mmHg and <95 mmHg and a SBP <155 mmHg, were randomly assigned to one of three groups. For 12 weeks, the placebo group received barley extract without catechin; another group received “Sunrouge” extract containing 11.2 mg anthocyanin and 323.6 mg epigallocatechin-3-*O*-gallate (EGCG); and a third group received “Yabukita” extract containing 322.2 mg EGCG. Home BP, accommodation ability, visual analog scale questionnaires for eyestrain, and metabolic-associated markers were analyzed at weeks 0, 4, 8, and 12 of the intake period. The ingestion of “Sunrouge” tea significantly improved accommodation ability and eyestrain in subjects younger than 45 years and in subjects who operated visual display terminals every day. It also elevated BP. “Yabukita” tea ingestion significantly increased serum adiponectin levels. No adverse effects were observed. We conclude that long-term intake of “Sunrouge” tea containing anthocyanins and flavonols might improve visual function.

## 1. Introduction

In recent years, fatigue and eyestrain associated with the use of visual display terminals (VDT) have become increasingly prevalent among younger individuals. In Japan, the penetration ratios of personal computers and smartphones in households reached 76.8% and 72.0%, respectively, in 2015 [1], while the ownership rates of mobile phones reached 128.0% in 2016 [2]. Longer VDT operation times result in increased stress and fatigue in different body regions, such as tired eyes and eye pain (91.6%), stiffness in the shoulders and neck (70.4%), and lower back pain (26.6%) [3]. Eyestrain is increasing due to long hours of VDT operation and its consequences, such as sleep deprivation or mental stress. It has been reported that approximately 90% of the Japanese population has eyestrain, making it one of the factors that lowers their quality of life (QOL) [3]. Research and drug development for eyestrain are scarce; functional foods are expected to contribute to the treatment of eyestrain.

Green tea (*Camellia sinensis* L.) is consumed worldwide, with large quantities being consumed in Japan and China, where it has been used for medicinal purposes for centuries. Green tea contains many functional ingredients such as catechins, caffeine, flavonols, vitamins, and dietary fiber. It has been reported to have various bioregulatory functions, such as anti-carcinogenic and anti-metastatic [4], anti-obesity [5], anti-diabetes [6], anti-hypercholesterolemic [7,8], antioxidative [9], anti-cardiovascular [10], anti-allergic [11], and anti-dry-eye activities [12], and it is known to improve the intestinal flora. The tea cultivars (cv.) “Yabukita” and “Sunrouge” [13], used as test samples in this study, contain catechins, including epigallocatechin-3-*O*-gallate (EGCG), which are believed to prevent lifestyle-related diseases. In addition, “Sunrouge” tea is rich in antioxidative anthocyanins [14,15,16]. It has been reported that tea catechins have an anti-hypertensive effect [17,18], while anthocyanins are well known to reduce eyestrain [19,20]. “Sunrouge” water extract suppressed acetylcholinesterase activity in human neuroblastoma SK-N-SH cells [15]. Neostigmine methyl sulfate, which is an acetylcholinesterase inhibitor, is used as an eye-focusing medicine [21].

Therefore, in this study, to clarify the effects of ingesting “Yabukita” green tea and “Sunrouge” green tea on eyestrain and hypertension, we conducted a 12-week, randomized, double-blind, placebo-controlled trial in Japanese adults (aged 20–60 years) with slightly elevated blood pressure (BP).

## 2. Materials and Methods

### 2.1. Study Design

**Figure 1:**
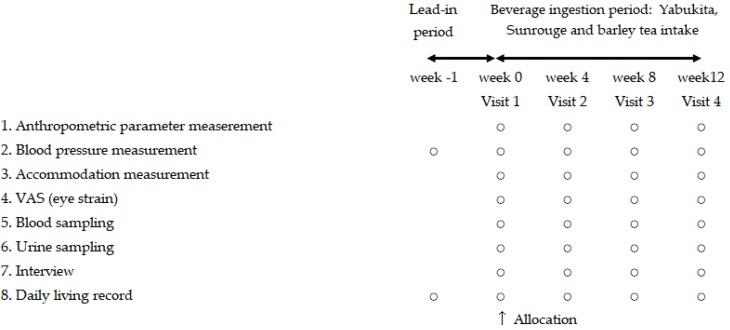
Schematic representation of the study/protocol.

This was a double-blind, randomized, placebo-controlled study. The study protocol is shown in Figure 1. The subjects were recruited by the Hokkaido Information University and were fully informed regarding the content and methods of this study. Written informed consents were obtained from subjects before their enrollment. The screening for the first volunteers started in July 2015 and the study was completed in January 2016. 

### 2.2. Subjects

We recruited a total of 258 healthy volunteers (aged 21–55 years), of which 120 subjects (50 men, 70 women) were eligible. Key eligibility and exclusion criteria are shown in Table S1. The 120 eligible subjects were randomly assigned to the “Yabukita” group, “Sunrouge” group, or placebo group stratified by sex, age, hospital-measured SBP, and eye strain severity score. Assignments were computer-generated using stratified block randomization at a third-party data center. Doctors, nurses, clinical research coordinators, and statistical analyzers had no knowledge of the assignment information during this trial period. This information was only disclosed after the laboratory and analytical data were fixed and the method of statistical analysis was finalized.

### 2.3. Test Samples

Three types of test drinks were manufactured industrially by Nikken Foods Co. Ltd. (Tokyo, Japan). Green tea (cv. “Yabukita” and “Sunrouge”) was extracted in hot water and the tea extract was concentrated and spray-dried to prepare a composite granulated powder with cyclodextrin. Barley tea extract powder that included no catechins and caffeine was used as the placebo. Subjects drank the tea, obtained by diluting the extract powder at the time of drinking, after each meal. The extracts were analyzed for flavonoids, caffeine, and anthocyanin content by high-performance liquid chromatography using UV-VIS detection [15,22]. The analytical test results are summarized in Table 1.

### 2.4. Study Outcomes

The primary outcomes measured were systolic blood pressure (SBP), diastolic blood pressure (DBP), accommodation (focusing ability of the eye) after stress or after rest, and the difference between accommodation ability after rest and after stress. 

Secondary outcomes measured were answers to a visual analog scale (VAS) questionnaire for eyestrain symptoms, intraocular pressure, peripheral blood flow, oxidized low density lipoprotein (Ox-LDL), thiobarbituric acid reactive substances (TBARS), superoxide dismutase (SOD), 8-hydroxy-2′-deoxyguanosine (8-OHdG), high sensitivity-C-reactive protein (hs-CRP), adiponectin, asymmetric dimethylarginine (ADMA), total homocysteine, and low density lipoprotein cholesterol (LDL-C).

### 2.5. Measurement of Blood Pressure

The subjects performed twice-daily home BP checks by themselves on the upper part of the arm opposite to the dominant hand, using an automated sphygmomanometer. BP measurement was carried out after urination and before breakfast within one hour after getting up in the morning, and after urination and before going to bed in the evening. Measurements were taken three times, with subjects in the sitting position, with five minutes of rest between measurements. The average of two stable measurement values was assumed to be the day’s measurement. BP values were determined before intake and at 4, 8, and 12 weeks after intake and were calculated as the averages of the BP measurements executed for 5 days prior to the recorded date of measurement. 

### 2.6. Measurement of Accommodation after VDT Work Load

To evaluate the effects of tea on eye focusing ability, we measured accommodation using an accommodate (D’ACOMO, WOC, Kyoto, Japan). Briefly, we asked subjects to press the stop button when the cross mark was clearly visible (their far point). Then, subjects were again asked to press the stop button when the cross mark became blurry (their near point). Each subjects’ amplitude of accommodation was measured by the distance between their near point and their far point; the resulting distance (cm) was converted into diopters (D). An increase in the value of D was considered to be an improvement in accommodation.

We asked the subjects to play the game “Tetris” using a tablet-type device for 20 min to induce eyestrain (stress period) and then to wear a blindfold during a 10 min break (break period). Accommodation of both of their eyes was monitored twice—once after the stress period and once after the break period. In addition, we asked them to ingest the test food right before the visual load of VDT work. 

We analyzed three kinds of accommodation values for each eye: accommodation measured after the stress period, the break period, and the difference between the stressing and the break time.

### 2.7. Measurement of Eyestrain Symptoms

We examined changes in eyestrain symptoms before and after (4, 8, 12 weeks) the intake using a visual analog scale (VAS) test. Our test was labeled “I feel extreme eyestrain” (0) and “I have no eyestrain” (100) on the extreme ends of a 100 mm line. The VAS questionnaire for eyestrain symptoms also included labels for “tired eyes,” “blurred vision,” “weight of eyelid,” “an ache behind the eyes,” “red eyes,” “bleary eyes,” “dryness of eyes,” “stiff shoulder,” “lower back pain,” “moodiness,” and “heaviness of the head.”

### 2.8. Adverse Events

Subjects were instructed to record any subjective symptoms regarding body conditions in their diaries, which were submitted to the responsible physician at the beginning of the run-in period; at weeks 0, 4, 8, and 12 of the intake period; and at the end of the withdrawal period.

### 2.9. Ethics Committee

This study protocol was approved by the Ethics Committee of the Hokkaido Information University (Ebetsu, Hokkaido, Japan) in accordance with the principles of the Helsinki Declaration (approval date 22 June 2015; approval number: 2015-08). This study was registered at www.umin.ac.jp/ctr/index.htm (registration number: UMIN000018469, date of registration 29 July 2015).

### 2.10. Statistical Analysis

The statistical analysis was performed using the per protocol analysis set. Values are expressed as means ± standard deviations (SD). Differences between intervention groups were evaluated by one-way ANOVA and the Tukey post-hoc tests. For each group, differences between pre- and post-intake were evaluated using paired *t*-tests. All statistical analyses were performed with SPSS ver. 19 (IBM Japan, Tokyo, Japan). 

A *p*-value of <0.05 was considered significant, while a *p*-value of <0.10 was defined as a marginal difference.

## 3. Results and Discussion

### 3.1. Number of Subjects and Intake Rate of Test Teas

**Figure 2:**
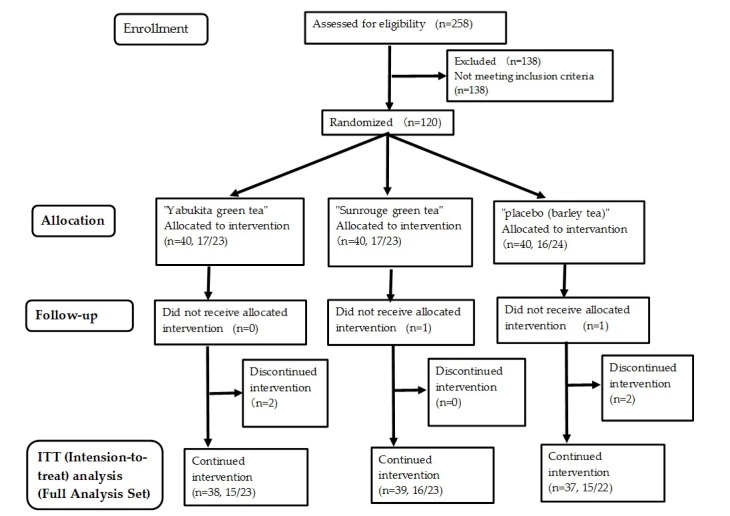
Flowchart of the study (*n* = total participants, male/female).

Thirty-eight subjects in the “Yabukita” group, 39 in the “Sunrouge” group, and 37 in the placebo group completed the final visit evaluation, and the data of these subjects were used for the final analysis. The average intake rates for the experimental period were 98.0 ± 3.6%, 97.7 ± 3.6%, and 99.4 ± 1.4%, as calculated using the intake check sheets of the “Yabukita” group, “Sunrouge” group, and placebo group, respectively. The flow diagram for this study is shown in Figure 2. The food survey using the food frequency questionnaire showed no change in the diet during the test period in either group (Table S2).

### 3.2. Physical Characteristics

The mean age, height, body weight, body mass index (BMI), and body fat ratio for each group are presented in Table 2. These data did not significantly differ among the three groups, confirming the appropriate allocation of subjects to the three groups. 

### 3.3. Primary Outcomes 

#### 3.3.1. Accommodation Ability (AA)

**Figure 3:**
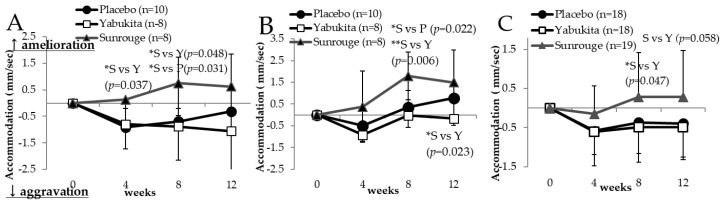
The changes in accommodation ability from week 0 to weeks 4, 8 and 12: (**A**), changes in accommodation ability of the left eye after a rest (under 45 years); (**B**) changes in accommodation ability of the left eye—the difference between the stressing and the break time (under 45 years); (**C**) changes in accommodation ability of the left eye after a rest (visual display terminals (VDT) operation). Statistical significance: * *p* < 0.05, ** *p* < 0.01.

The intraocular pressures of the right and left eye gradually increased in all test groups (Table S3). The intention to treat (ITT) analysis did not show an improvement of AA in the green tea groups. We performed a stratified analysis for age, because eye AA could not be measured accurately in the subjects with presbyopia. Changes in the AA of the left eye after rest in non-presbyopic subjects younger than 45 years at weeks 4 and 8 significantly improved in the “Sunrouge” group (*n* = 8) compared to the placebo group (*n* = 10) (Figure 3A). Changes in the AA of the left eye after rest and after stress in non-presbyopic subjects younger than 45 years at week 8 significantly improved in the “Sunrouge” group compared to the placebo group (Figure 3B). 

Changes in the left eye’s AA after rest in non-presbyopic subjects younger than 45 years of age at week 8 (*p* = 0.031) significantly improved in the “Sunrouge” group compared to the “Yabukita” group (Figure 3A). Changes in the difference between AA after rest and AA after stress of the left eye in non-presbyopic subjects younger than 45 at weeks 8 and 12 significantly improved in the “Sunrouge” group compared to the “Yabukita” group (Figure 3B).

At week 8, changes in the AA of the left eye after rest in subjects operating VDT every day significantly improved in the “Sunrouge” group compared to the “Yabukita” group (Figure 3C). 

In a stratified analysis of AA, significant improvement of left eye AA was observed in subjects younger than 45 years and in subjects operating VDT every day. A possible explanation for these results is the small difference between the near and far point, leading to an inaccurate evaluation of the AA. As for AA improvement being observed only for the left eye, it is thought that this is related to eye dominance, and needs further examination. Moreover, it is necessary to measure the AA before rest and consider this parameter to evaluate the improvement of AA. Improvement of accommodation ability by the “Sunrouge” group was also sufficient compared to other functional foods known to improve eyestrain; thus, we consider “Sunrouge” as a sufficiently valuable tea to improve eyestrain. 

#### 3.3.2. Home Blood Pressure

Morning SBP values at weeks 8 and 12, morning DBP values at weeks 0, 4, 8, and 12, and evening DBP values at weeks 4, 8, and 12 were significantly higher in the “Sunrouge” group than in the placebo group, but remained within the normal range for the Japanese population (SBP <140 and DBP <85), as shown in Table 3.

**Figure 4:**
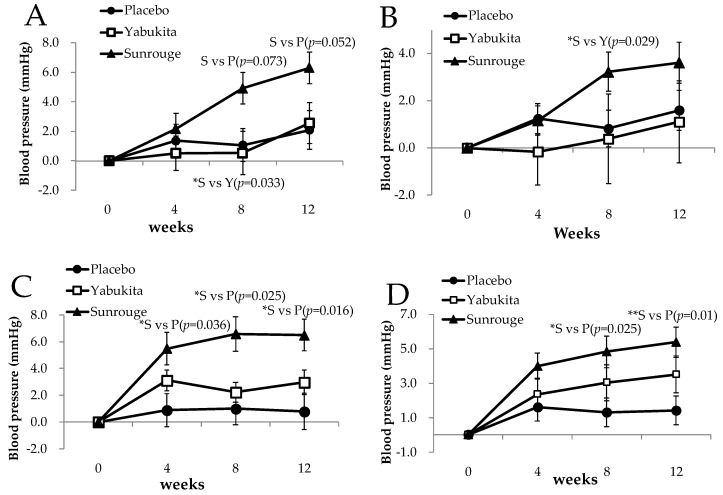
The changes in home-measured blood pressure in the morning and evening from week 0 to weeks 4, 8, and 12: (**A**) SBP change at waking up; (**B**) DBP change at waking up; (**C**) SBP change at bedtime; (**D**) DBP change at bedtime. Statistical significance: * *p* < 0.05, ** *p* < 0.01.

The evening SBP change at weeks 4, 8, and 12, and DBP change at weeks 8 and 12 were significantly higher in the “Sunrouge” group than in the placebo group, as shown in Figure 4C,D. The morning SBP change at week 12 was higher in the “Sunrouge” group than in the placebo group, though the difference was not statistically significant (*p* = 0.052) (Figure 4A). Moreover, the morning DBP and morning SBP changes at week 8 were significantly higher in the “Sunrouge” group than in the “Yabukita” group (Figure 4A,B). “Sunrouge” strongly raised blood pressure, though within the normal range for the Japanese population; thus, we consider this result clinically relevant. In this study, long-term ingestion of “Sunrouge” containing anthocyanins raised blood pressure, suggesting that “Sunrouge” may be usable as a hypertensive agent.

Several studies have reported that consecutive intake of 64 mg of anthocyanins by patients after cardiac infarction lowered BP, due to anthocyanins’ ameliorating effect on hypertension [23,24]. We hypothesized that BP regulation by anthocyanins is related to its antioxidative effect or improvement of the peripheral blood flow. However, the definite improvement of antioxidative markers or peripheral blood flow was not observed with “Sunrouge” ingestion in this study (Table S3); therefore, further investigation is needed to elucidate the relationship between “Sunrouge” intake and BP. Moreover, it is necessary to investigate the effect of “Sunrouge” on BP in subjects who are sensitive to cold temperature.

### 3.4. Secondary Outcomes

Secondary outcomes were data on intraocular pressure, peripheral blood flow, Ox-LDL, TBARS, SOD, 8-OHdG, hs-CRP, adiponectin, ADMA, total homocysteine, and LDL-C. Among oxidative stress markers, at week 4, TBARS and SOD levels in the “Yabukita” group were significantly higher than in the placebo group, while Ox-LDL and urinary 8-OHdG did not differ between the groups, as shown in Table 4 and Table S3.

### 3.5. Eyestrain Symptoms

We compared the changes listed in the VAS questionnaire for eyestrain symptoms—“tired eyes,” “blurred vision,” “weight of eyelid,” “an ache behind the eyes,” “red eyes,” “bleary eyes,” “dryness of eyes,” “stiff shoulder,” “lower back pain,” “moodiness,” and “heaviness of the head”—from baseline for each week in all test groups. We observed no significant differences among the three groups; therefore, a stratified analysis was conducted for the presence or absence of daily VDT operation and age.

**Figure 5:**
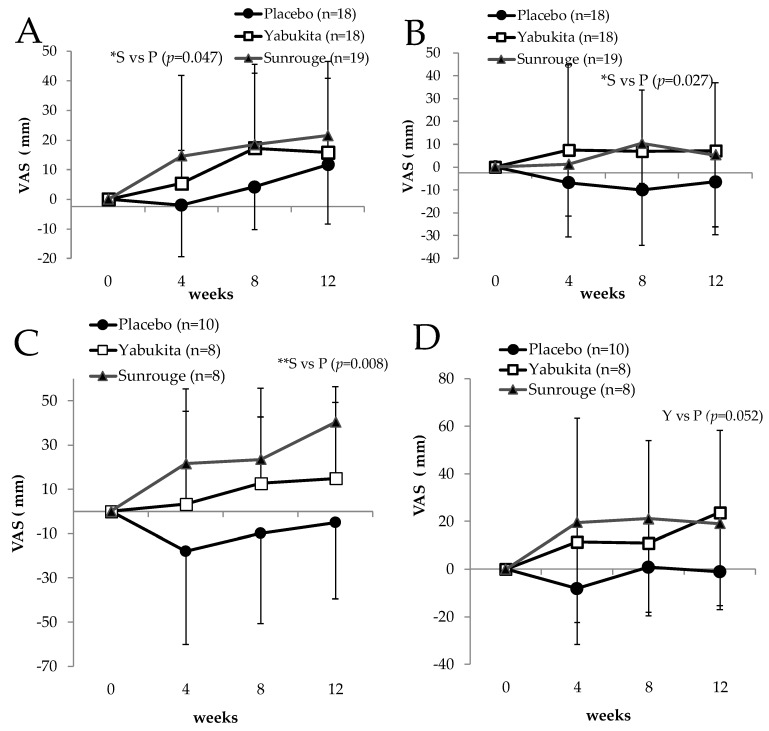
The changes in eyestrain symptoms from weeks 0 to weeks 4, 8 and 12: (**A**) changes in eye strain (subjects who do VDT operations everyday); (**B**) changes in lower back pain (subjects who do VDT operations everyday); (**C**) changes in top-heavy feeling (under 45 years); (**D**) changes in ache behind the eyes (under 45 years). Statistical significance: * *p* < 0.05, ** *p* < 0.01 vs. placebo group.

As shown in Figure 5, in subjects who performed VDT operations (PC, smartphone, tablet) everyday (placebo group: 18 people, “Yabukita” group: 19, “Sunrouge” group: 18), changes in eyestrain at week 4 (Figure 5A), and changes in lower back pain at week 8 (Figure 5B) significantly improved in the “Sunrouge” group compared to the placebo group. Moreover, for subjects younger than 45 years (placebo group: 10 people, “Yabukita” group: 8, “Sunrouge” group: 8), the “Sunrouge” group showed significant improvement in the top-heavy feeling at week 12 compared to those in the placebo group (Figure 5C). Subjects younger than 45 years in the “Yabukita” group experienced less ache behind the eyes at week 12 compared to those in the placebo group, though the difference was not statistically significant (*p* = 0.052) (Figure 5D). 

The biological mechanism of the eye strain reduction effect of anthocyanins is thought to be the effect of antioxidants [25] and rhodopsin regeneration [26]. The above results suggest that the eyestrain-reducing effect of “Sunrouge” was higher in subjects performing many VDT operations. Because changes in the top-heavy feeling significantly improved in the “Sunrouge” group in subjects younger than 45 years, the eyestrain-reducing effect of Sunrouge may be higher in subjects who do not have presbyopia. 

A similar result has been reported for blackcurrant anthocyanin. Nakanishi et al. reported that after the intake of 50 mg of blackcurrant anthocyanin concentrate (delphinidin-3-rutinoside; 4.6%, delphinidin-3-glucoside; 1.4%, cyanidin-3-rutinoside; 2.8%, cyanidin-3-glucoside; 0.4%), significant improvement was seen in eyestrain symptoms (eye and lower back), assessed using a questionnaire [19]. Lee et al. reported that the administration of anthocyanoside oligomer (85 mg) seemed to improve subjective symptoms and objective contrast sensitivity in subjects with myopia and asthenopia [20].

In the future, a Randomized Controlled Trial (RCT) needs to be carried out on the effect of “Sunrouge” green tea on the reduction of eyestrain in non-presbyopic subjects with a workload involving increased VDT operation. No blood flow improvement was associated with “Sunrouge” intake, as assessed by peripheral blood flow measurement, but these results do not exclude the possibility that consecutive intake of “Sunrouge” tea, which rich in anthocyanins, may have positive effects on blood flow. Therefore, further examination is needed on blood flow improvement by “Sunrouge” tea.

### 3.6. Endothelial Markers

**Figure 6:**
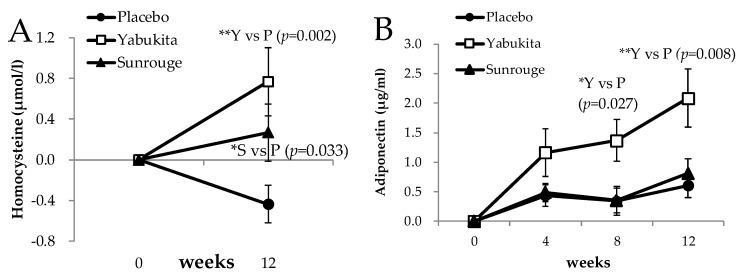
The changes in homocysteine and adiponectin levels from weeks 0 to weeks 4, 8, and 12: (**A**) change in homocysteine; (**B**) change in adiponectin. Statistical significance: * *p* < 0.05, ** *p* < 0.01 vs. placebo group.

Changes in serum homocysteine were significantly higher in the “Yabukita” and “Sunrouge” groups at week 12 than in the placebo group, as shown in Figure 6A. The changes in serum adiponectin levels were significantly higher in the “Yabukita” group at weeks 8 and 12 than in the placebo group (Figure 6B). It has been reported that dietary supplementation with 0.25% EGCG elevated levels of circulating adiponectin in non-obese type-2 diabetic Goto-Kakizaki rats [27]. Moreover, there was a significant increase in plasma adiponectin levels and plasma LDL particle size in subjects with coronary artery disease after one-month intake of oolong tea containing 245.6 mg of polyphenols [28]. Green tea catechin administration to rats with high-fat, diet-induced obesity increased the levels of peroxisome proliferator-activated receptor (PPAR)γ in subcutaneous white adipose tissue and decreased those of PPARγ in visceral white adipose tissue [29]. 

In the present study, the changes in serum adiponectin significantly increased in the group administered “Yabukita” containing 322 mg of EGCG. Although “Sunrouge” green tea contained as much EGCG and caffeine as “Yabukita” green tea, the change in serum adiponectin did not increase upon the intake of “Sunrouge” green tea. “Sunrouge” green tea contains epigallocathin-3-*O*-(3-*O*-methyl)-gallate, one-fifth the epigallocatechin, twice the myricetin, two-fifths of the quercetin and a seven-tenths of the kaempferol of “Yabukita” green tea (Table 1). We hypothesize that this difference is due to a difference in the composition ratios of anthocyanins, catechins, or flavonols, rather than to differences in the EGCG content between “Sunrouge” green tea and “Yabukita” green tea. However, it is not clear if caffeine was driving any of these effects, because caffeine was absent from the placebo group.

A subgroup analysis was performed between the two subject groups with and without a daily habit of tea drinking (*n* = 12 and 13 for daily tea drinkers in the placebo and green tea groups, respectively; *n* = 25 for non-daily tea drinkers in both the placebo group and green tea groups). The change in HDL cholesterol levels from week 0 to week 12 in the “Yabukita” tea group (5.28 ± 9.11 mg/dL) was significantly higher than that in the placebo group (−1.00 ± 6.81 mg/dL) (*p* = 0.008). This result suggests that the effect of “Yabukita” tea on lipid metabolism is greater in subjects without a daily habit of tea drinking. 

In this study, “Yabukita” tea did not affect BP, antioxidant actions, endothelial markers, or peripheral blood flow. However, serum adiponectin and HDL-C content were significantly higher in members of the “Yabukita” group who did not have a daily habit of tea drinking. It will be necessary to evaluate the efficacy of “Yabukita” tea in improving lipid metabolism or against body fat accumulation in subjects with a slightly increased cholesterol level or BMI in the future.

### 3.7. Safety and Hospitalization

We evaluated complete blood cell counts (CBCs), liver and renal function, and BP after the ingestion of tea. As shown in Table S2, minimal changes were observed in CBC parameters (white blood cell (WBC) count, red blood cell (RBC) count, hemoglobin (Hb) level, hematocrit (Ht), and platelet (Plt) count). Changes were also minimal for liver function (aspartate aminotransferase (AST), alanine aminotransferase (ALT), γ-glutamyl transpeptidase (γ-GTP), alkaline phosphatase (ALP), and lactate dehydrogenase (LDH)), renal function (urea nitrogen (BUN), creatinine (CRE), and uric acid (UA)), albumin (Alb), total protein (TP), unsaturated iron binding capacity (UIBC), and total iron binding capacity(TIBC). 

One subject who ingested the placebo tea exhibited an adverse event related to liver function, and recovered from this symptom within a week. One subject who ingested the “Yabukita” tea was admitted to the hospital because of an acute virus infection. An additional subject who ingested the “Yabukita” tea was admitted to the hospital with venous thrombosis; since the onset of the disease occurred immediately after the start of the study, this subject had likely not ingested the test meal. The relationship between homocysteine and thrombus formation was higher in “Yabukita” group; however, the values of all subjects were in the normal range. Accordingly, the principal investigator judged that there were no serious adverse events related to the study intervention. These results suggest that the intake of “Sunrouge” or “Yabukita” tea containing 324 mg of EGCG, 203 mg of caffeine and 11 mg of anthocyanins for 12 weeks is safe. “Sunrouge” and “Yabukita” teas have large amounts of catechins including 300 mg of EGCG, and it has been reported that a large amount of EGCG may cause hepatic damage [30], but 704mg EGCG per day might be considered safe, based on observed human adverse event data [31]. However, we should carefully watch our intake of catechins.

## 4. Conclusions

Consecutive ingestion of the “Sunrouge” tea extract significantly improved eye AA in subjects younger than 45 years and in subjects operating VDT every day. It also elevated BP. Moreover, in subjects who operated VDT everyday, the “Sunrouge” group noted significant improvements in eyestrain at week 4, lower back pain at week 8, and top-heavy feelings at week 12 when compared to the placebo group. This result shows the possibility that “Sunrouge” is usable as an eyestrain-reducing agent or a hypertensive agent. “Yabukita” tea extract ingestion significantly increased serum adiponectin levels. No adverse effects were observed. These results suggest that the eyestrain-reducing effect of “Sunrouge” is higher in subjects younger than 45 years or in subjects operating VDT. The eyestrain-reducing effect of “Sunrouge” may therefore be higher in subjects who do not have presbyopia. In the future, we need to carry out an RCT on the eyestrain-reducing effect of “Sunrouge” tea for non-presbyopic subjects with a workload involving increased VDT operation. We hypothesize that the difference in efficacy is due to a difference in anthocyanins, catechins, or flavonols composition ratios rather than a difference in EGCG content between “Sunrouge” green tea and “Yabukita” green tea.

## Figures and Tables

**Table 1:** Functional components in test teas (mg/day).

	“Yabukita” Green Tea	“Sunrouge” Green Tea	Placebo Barley Tea
Anthocyanins	0.0	11.2 *	0.0
EGCG ^2^	322.2	323.6	0.0
EGC ^3^	413.1	85.7	0.0
EGCG3”Me ^4^	0.0	12.3	0.0
Flavonols	54.8 ^5^	40.4 ^6^	0.0
Caffeine	202.5	184.8	0.0

* delphinidin glucosides, 7.9 mg; cyanidin glucosides, 3.3mg; ^2^ EGCG, epigallocatechin-3-*O*-gallate; ^3^ EGC, epigallocatechin; ^4^ EGCG3”Me, epigallocatchin-3-*O*-(3-*O*-methyl)-gallate; ^5^ myricetin glucosides, 11.7 mg; quercetin glucosides, 32.2 mg; kaempferol glucosides, 10.9 mg; ^6^ myricetin glucosides, 20.1 mg; quercetin glucosides, 13.4 mg; kaempferol glucosides, 6.9 mg.

**Table 2:** Baseline characteristics of subjects taking the test teas “Yabukita”, “Sunrouge”, or the placebo barley tea.

Baseline Characteristics	“Yabukita”	“Sunrouge”	Placebo
Gender(male/female) (*n*)	38 (15/23)	39 (16/23)	37 (15/22)
Age (years)	49.8 ± 6.7	49.5 ± 6.2	48.5 ± 7.5
Body weight (kg)	64.3 ± 9.7	61.4 ± 11.7	62.1 ± 10.0
Body mass index (kg/m^2^)	23.9 ± 3.3	23.3 ± 3.4	23.3 ± 2.4
Body fat ratio (%)	28.9 ± 6.9	27.9 ± 6.3	28.7 ± 6.7
Home SBP at waking up (mmHg)	126.8 ± 11.9	126.6 ± 14.2	124.6 ± 10.1
Home DBP at waking up (mmHg)	80.5 ± 9.8	81.8 ± 9.8 *	77.3 ± 7.4
Home SBP at bedtime (mmHg)	123.7 ± 11.9	123.3 ± 12.9	123.7 ± 11.0
Home DBP at bedtime (mmHg)	75.3 ± 10.0	77.1 ± 8.5	74.8 ± 7.0
Pulse (bpm)	74.6 ± 9.9	75.8 ± 11.1	74.4 ± 10.7

Values are shown as the means ± standard deviations. *n* = number of subjects; SBP, systolic blood pressure; DBP, diastolic blood pressure; One-way ANOVA and the Tukey post-hoc test vs. placebo; paired *t*-test vs. 0 week was performed to analyze the values. Statistical significance: * *p* < 0.05 vs. placebo group.

**Table 3:** Changes in home blood pressure after drinking “Sunrouge”, “Yabukita”, or barley infusion tea.

	Interventions	Week 0	Week 4	Week 8	Week 12
Home SBP at waking up	“Yabukita”	126.8 ± 11.9	127.3 ± 11.9	127.3 ± 13.8	129.3 ± 14.4
(mmHg)	“Sunrouge”	126.6 ± 14.2	128.8 ± 13.5 ^#^	131.5 ± 13.4 *^##^	132.9 ± 14.3 *^##^
	Placebo	124.6 ± 10.1	125.2 ± 9.4	125.6 ± 9.2	126.7 ± 10.5
Home DBP at waking up	“Yabukita”	80.5 ± 9.8	80.3 ± 8.7	80.8 ± 9.7	81.6 ± 1.6
(mmHg)	“Sunrouge”	81.8 ± 9.8 *	83.0 ± 8.7 *	85.1 ± 8.8 **^##^	85.5 ± 1.5 **^##^
	Placebo	77.3 ± 7.4	78.4 ± 7.0	78.1 ± 7.7	78.9 ± 1.4
Home SBP at bedtime	“Yabukita”	123.7 ± 11.9	126.8 ± 13.5	125.9 ± 15.6	126.3 ± 14.7
(mmHg)	“Sunrouge”	123.3 ± 12.9	128.8 ± 13.8 ^##^	129.9 ± 13.7 ^##^	129.8 ± 12.7 ^##^
	Placebo	123.7 ± 11.0	123.8 ± 13.8	124.7 ± 10.5	124.5± 12.6
Home DBP at bedtime	“Yabukita”	75.3 ± 10.0	77.7± 10.3	78.4 ± 10.9	78.8 ± 11.1
(mmHg)	“Sunrouge”	77.1 ± 8.5	81.0 ± 8.9 *^##^	81.9 ± 8.5 **^##^	82.4 ± 8.2 **^##^
	Placebo	74.8 ± 7.0	76.3 ± 7.3	76.1 ± 8.7	76.2 ± 9.0

Values are shown as the means ± standard deviations; SBP, systolic blood pressure; DBP, diastolic blood pressure; One-way ANOVA and the Tukey post-hoc test vs. placebo, paired *t*-test vs. 0 week were performed to analyze the values. Statistical significance: * *p* < 0.05, ** *p* < 0.01 vs. placebo group, ^#^ *p* < 0.05, ^##^ *p* < 0.01 vs. 0 week.

**Table 4:** Secondary outcomes after drinking “Sunrouge”, “Yabukita”, or barley infusion.

	Interventions	Week 0	Week 4	Week 8	Week 12
Oxidation marker					
TBARS (mM)	“Yabukita”	13.7 ± 3.5	16.2 ± 5.4 *^#^	9.9 ± 3.3 ^##^	12.8 ± 3.8
	“Sunrouge”	13.0 ± 3.3	13.9 ± 5.3	9.1 ± 3.5 ^##^	12,0 ± 4.0
	Placebo	13.8 ± 3.7	14.2 ± 3.9	8.5 ± 3.0 ^##^	11.9 ± 3.5 ^#^
SOD (U/L)	“Yabukita”	3.2 ± 1.8	2.7 ± 1.8 *^#^	2.9 ± 1.9 *^##^	2.7 ± 2.2 ^##^
	“Sunrouge”	3.0 ± 1.6	2.4 ± 1.6 ^##^	2.9 ± 2.2	2.6 ± 2.1 ^##^
	Placebo	2.6 ± 0.8	2.0 ± 0.8 ^##^	2.3 ± 1.2 ^#^	2.1 ± 1.3 ^##^

Values are shown as the means ± standard deviations; TBARS, 2-thiobarbituric acid reactive substances, SOD, superoxide dismutase. Statistical significance: * *p* < 0.05, ** *p* < 0.01 vs. placebo group, ^#^ *p* < 0.05, ^##^ *p* < 0.01 vs. 0 week.

## Supplementary Materials

The following are available online at http://www.mdpi.com/2072-6643/10/5/569/s1, Table S1. Key eligibility and exclusion criteria, Table S2. Changes in the anthrometric values and biochemical parameters after drinking “Sunrouge”, “Yabukita”, or barley tea, Table S3. Secondary outcomes after drinking “Sunrouge”, “Yabukita”, or barley tea.
