# Supplementary material for: A Randomized, Placebo-Controlled Study on the Safety and Efficacy of Daily Ingestion of Green Tea (Camellia sinensis L.) cv. “Yabukita” and “Sunrouge” on Eyestrain and Blood Pressure in Healthy Adults
Source: Nutrients. 2018 May 6;10(5):569. doi: 10.3390/nu10050569 (PMC5986449; doi:10.3390/nu10050569)
Supplement: Supplementary file 1 [file nutrients-10-00569-s001.pdf]

nutrients

MDPI

Article

Table S1. Key eligibility and exclusion criteria

Eligibility criteria;

1. Hospital systolic blood pressure (SBP) is 130 mmHg or more but less than 160 mmHg and diastolic blood pressure (DBP) is less than 100 mmHg
2. Hospital DBP 80 is mmHg or more but less than 100 mmHg and SBP is less than 160 mmHg
3. Home SBP is 125 mmHg or more but less than 155 mmHg and DBP is less than 95 mmHg
4. Home DBP is 75 mmHg or more but less than 95 mmHg and SBP is less than 155 mmHg

Key exclusion criteria;

1. Subjects who are under treatment and medication for hypertension.
2. Subjects who are under treatment and medication for ocular disease (except for myopia, astigmatism, hyperopia, presbyopia, dry eye, and allergic conjunctival disease) or having serious ocular diseases.
3. Subjects who have a major ophthalmic surgical history such as cataract, glaucoma, refractive correction.
4. Subjects with serious cerebrovascular, cardiac, hepatic, renal, and/or gastrointestinal diseases, or affected with an infectious disease which is required to be reported to the authorities.
5. Subjects who have a major surgical history related to the digestive system such as gastrectomy.
6. Subjects with unusually high and/or low BP or abnormal hematological data.
7. Subjects with serious anemia.
8. Pre- or post-menopausal subjects having complaints of obvious physical changes.
9. Subjects who are at risk of having allergic reactions to drugs or foods (especially green tea).
10. Subjects who have high caffeine sensitivity.
11. Subjects who regularly take medicine, functional foods and/or supplements (peptide, acetic acid, gamma-aminobutyric acid (GABA), geniposidic acid, flavonoids, sesamin, chlorella, olive leaves, garlics, dietary fiber, tomatoes, folic acid, etc.) that would affect the BP.
12. Subjects who regularly take medicine, functional foods and/or supplements (anthocyanin, lutein, astaxanthin, blueberry, beta-carotene, vitamin, docosahexaenoic acid (DHA), eicosapentaenoic acid (EPA) etc.) that would affect the visual fatigue.
13. Heavy smokers, alcohol addicts or subjects with irregular lifestyles.
14. Subjects who donate either 400 ml of whole blood within 12 weeks or 200 ml of whole blood within 4 weeks or blood components within 2 weeks prior to the start of this study.
15. Subjects who are pregnant or expected to be pregnant, or lactating during the study.
16. Subjects who participated in other clinical trials within the last month prior to the current clinical trial.
17. Any other medical reasons judged by the principal investigator.

Table S2. Changes in the anthrometric values and biochemical parameters after drinking "Sunrouge", "Yabukita", or barley tea

	Interventions	Week 0	Week 4	Week 8	Week 12
Body mass index	"Yabukita"	23.9 ± 3.3	24.0 ± 3.3	24.1 ± 3.2	24.0 ± 3.1
(kg/m^2)	"Sunrouge"	23.3 ± 3.4	23.5 ± 3.4	23.5 ± 3.5	23.5 ± 3.5
	Placebo	23.3 ± 2.4	23.6 ± 2.5	23.6 ± 2.5	23.6 ± 2.4
Body fat ratio (%)	"Yabukita"	28.9 ± 6.9	29.8 ± 7.2	29.7 ± 7.2	29.9 ± 7.1
	"Sunrouge"	27.9 ± 6.3	28.8 ± 6.2	28.6 ± 6.1	28.4 ± 6.2
	Placebo	28.7 ± 6.7	29.8 ± 6.3	29.5 ± 6.5	29.6 ± 6.2
Pulse (bpm)	"Yabukita"	74.6± 9.9	75.4 ± 9.5	75.5 ± 11.1	76.4 ± 12.1
	"Sunrouge"	75.8 ± 11.1	73.2 ± 11.8	74.3 ± 12.6	76.5 ± 11.3
	Placebo	74.4 ± 10.7	73.1 ± 9.9	76.2 ± 12.4	77.0 ± 12.6
Lipid parameter					
Total cholesterol (mg/dL)	"Yabukita"	220.1 ± 41.1	223.3 ± 40.7	220.1 ± 42.7	224.1 ± 42.8
	"Sunrouge"	223.4 ± 32.0	219.6 ± 32.6	213.1 ± 35.1 #	213.5 ± 31.3 ##
	Placebo	221.1 ± 32.7	220.3 ± 29.8	213.5 ± 30.2 #	215.0 ± 35.3
HDL-C (mg/dL)	"Yabukita"	69.1 ± 21.7	72.4 ± 21.5 #	71.8 ± 22.6	73.3 ± 24.0 *#
	"Sunrouge"	72.7 ± 17.5 *	70.7 ± 15.6	70.0 ± 15.2 *#	71.4 ± 16.1 *
	Placebo	63.7 ± 14.7	64.9 ± 16.5	62.8 ± 16.1	63.4 ± 16.1
Triglyceride (mg/dL)	"Yabukita"	111.4 ± 68.2	104.3 ± 64.6	120.8 ± 88.7	107.5 ± 79.9
	"Sunrouge"	104.6 ± 63.9	114.7 ± 86.8	98.5 ± 52.9	103.3 ± 59.5
	Placebo	121.0 ± 86.6	135.9 ± 106.8 #	124.4 ± 96.6	125.0 ± 126.4
NEFA (mEq/L)	"Yabukita"	0.55 ± 0.19	0.49 ± 0.19 **	0.49 ± 0.20 **	0.53 ± 0.24 **
	"Sunrouge"	0.64 ± 0.18	0.48 ± 0.18 **##	0.47 ± 0.16 **##	0.49 ± 0.20 **##
	Placebo	0.60 ± 0.16	0.34 ± 0.14 ##	0.35 ± 0.13 ##	0.37 ± 0.15 ##
Glycometabolism associated parameters					
HbA1c (%)	"Yabukita"	5.4 ± 0.6	5.5 ± 0.7	5.5 ± 0.7	5.5 ± 0.7
	"Sunrouge"	5.4 ± 0.6	5.5 ± 0.6	5.5 ± 0.7	5.5 ± 0.7
	Placebo	5.3 ± 0.3	5.3 ± 0.3	5.4 ± 0.3	5.4 ± 0.3
Blood glucose (mg/dL)	"Yabukita"	90.3 ± 16.3	93.0 ± 18.3	93.1 ± 15.5	96.1 ± 19.3
	"Sunrouge"	90.6 ± 13.5	94.2 ± 16.9	95.8 ± 20.0	95.1 ± 18.7
	Placebo	90.2 ± 11.3	92.0 ± 10.9	91.6 ± 11.0	93.0 ± 11.1
Glycoalubulin (%)	"Yabukita"	14.6 ± 1.9	14.3 ± 2.0	14.0 ± 1.9	14.0 ± 1.9
	"Sunrouge"	14.8 ± 1.8	14.5 ± 2.0	14.2 ± 2.1	14.3 ± 2.0
	Placebo	13.9 ± 1.1	13.8 ± 1.1	13.5 ± 1.1	13.6 ± 1.1
Complete blood count					
WBC (x10^3/mL)	"Yabukita"	5.6 ± 2.1	5.5 ± 1.4	5.8 ± 1.7	5.3 ± 1.4
	"Sunrouge"	5.5 ± 1.5	5.7 ± 1.7	5.6 ± 1.7	5.9 ± 1.3
	Placebo	5.4 ± 1.8	5.5 ± 1.6	5.6 ± 2.1	5.2 ± 1.9
RBC (x10^4/mL)	"Yabukita"	476.6 ± 45.3	485.5 ± 46.5	477.8 ± 43.5	480.8 ± 43.7
	"Sunrouge"	469.7 ± 41.3	469.4 ± 42.8	467.1 ± 43.2	465.6 ± 43.6
	Placebo	461.0 ± 40.3	460.7 ± 35.8	460.0 ± 42.7	465.2 ± 45.1
Hb(g/dL)	"Yabukita"	14.1 ± 1.5	14.4 ± 1.5	14.1 ± 1.5	14.2 ± 1.6
	"Sunrouge"	14.1 ± 1.5	14.1 ± 1.5	14.0 ± 1.5	13.9 ± 1.5
	Placebo	13.9 ± 1.3	13.9 ± 1.2	13.9 ± 1.4	14.0 ± 1.4
Ht (%)	"Yabukita"	42.8 ± 4.1	44.0 ± 4.3	43.7 ± 4.2	43.5 ± 4.3
	"Sunrouge"	42.4 ± 3.6	42.9 ± 3.9	42.8 ± 3.9	42.5 ± 3.7
	Placebo	42.0 ± 3.4	42.4 ± 3.1	42.6 ± 3.6	42.7 ± 3.8
Plt (x10^4/mL)	"Yabukita"	23.3 ± 4.1	23.9 ± 3.9	24.6 ± 4.7	24.4 ± 4.4
	"Sunrouge"	23.8 ± 5.1	25.4 ± 5.4	25.9 ± 5.9	25.3 ± 5.3
	Placebo	24.1 ± 4.1	25.7 ± 4.6	25.3 ± 5.0	24.9 ± 4.3

Liver function					
AST (U/L)	"Yabukita"	21.5 ± 5.0	22.2 ± 5.2	23.7 ± 6.2	22.2 ± 4.7
	"Sunruge"	21.8 ± 5.2	22.8 ± 6.2	24.0 ± 8.7	23.4 ± 5.9
	Placebo	21.8 ± 6.9	22.4 ± 7.2	22.3 ± 7.1	21.1 ± 4.7
ALT (U/L)	"Yabukita"	21.7 ± 10.8	24.0 ± 12.8	25.1 ± 14.5	22.0 ± 10.7
	"Sunruge"	20.7 ± 9.0	22.9 ± 13.2	22.8 ± 12.4	23.2 ± 13.1
	Placebo	22.4 ± 14.3	23.4 ± 15.2	22.5 ± 15.9	21.2 ± 11.3
ALP (U/L)	"Yabukita"	193.8 ± 49.7	200.7 ± 55.7	199.9 ± 50.6	200.1 ± 46.9
	"Sunruge"	184.1 ± 57.4	197.8 ± 69.8	196.1 ± 61.8	198.0 ± 73.1
	Placebo	203.7 ± 56.0	211.0 ± 60.6	208.2 ± 57.2	209.0 ± 62.9
LDH (U/L)	"Yabukita"	189.7 ± 27.3	202.1 ± 24.3	211.0 ± 28.8	196.1 ± 24.1
	"Sunruge"	188.6 ± 38.0	201.8 ± 41.2	205.9 ± 32.7	196.0 ± 34.5
	Placebo	179.9 ± 22.8	190.5 ± 26.8	194.1 ± 30.6	183.1 ± 24.9
γGTP (mg/dL)	"Yabukita"	33.9 ± 29.6	39.9 ± 41.0	39.4 ± 42.0	36.7 ± 31.4
	"Sunruge"	27.5 ± 15.6	31.3 ± 19.7	30.1 ± 20.0	34.2 ± 41.4
	Placebo	34.6 ± 32.6	35.3 ± 33.3	35.7 ± 36.5	36.7 ± 44.7
LAP (U/L)	"Yabukita"	55.4 ± 10.9	57.7 ± 13.7	57.0 ± 12.5	56.1 ± 10.6
	"Sunruge"	53.4 ± 7.8	54.0 ± 9.6	53.4 ± 8.5	54.4 ± 10.7
	Placebo	55.6 ± 14.3	55.3 ± 14.5	55.3 ± 14.5	56.2 ± 20.0
ChE (U/L)	"Yabukita"	338.3 ± 76.7	362.0 ± 79.4	363.2 ± 86.3	368.1 ± 82.8
	"Sunruge"	334.1 ± 66.8	344.8 ± 74.6	344.0 ± 66.6	349.2 ± 70.1
	Placebo	334.1 ± 74.2	344.1 ± 75.4	340.5 ± 78.4	346.3 ± 79.1
ZTT (KU)	"Yabukita"	6.5 ± 2.3	6.1 ± 2.3	6.2 ± 2.4	6.2 ± 2.4
	"Sunruge"	6.8 ± 2.4	6.4 ± 2.4	6.5 ± 2.6	6.6 ± 2.4
	Placebo	7.2 ± 2.6	7.0 ± 2.7	7.1 ± 2.5	7.2 ± 2.4
TP (mg/dL)	"Yabukita"	7.2 ± 0.4	7.4 ± 0.3	7.3 ± 0.4	7.3 ± 0.4
	"Sunruge"	7.4 ± 0.4	7.5 ± 0.5	7.4 ± 0.4	7.4 ± 0.5
	Placebo	7.3 ± 0.4	7.4 ± 0.3	7.3 ± 0.4	7.3 ± 0.4
Albumin (mg/dL)	"Yabukita"	4.6 ± 0.3	4.7 ± 0.3	4.6 ± 0.3	4.6 ± 0.3
	"Sunruge"	4.7 ± 0.2	4.7 ± 0.2	4.6 ± 0.2	4.6 ± 0.3
	Placebo	4.6 ± 0.3	4.7 ± 0.2	4.6 ± 0.3	4.6 ± 0.2
A/G ratio	"Yabukita"	1.7 ± 0.2	1.8 ± 0.2	1.7 ± 0.2	1.8 ± 0.2
	"Sunruge"	1.7 ± 0.2	1.8 ± 0.2	1.7 ± 0.2	1.7 ± 0.2
	Placebo	1.8 ± 0.3	1.8 ± 0.2	1.7 ± 0.2	1.7 ± 0.2
T-Bil (mg/dL)	"Yabukita"	0.7 ± 0.3	0.7 ± 0.3	0.7 ± 0.2	0.7 ± 0.3
	"Sunruge"	0.8 ± 0.3	0.7 ± 0.3	0.7 ± 0.3	0.7 ± 0.3
	Placebo	0.8 ± 0.2	0.7 ± 0.3	0.6 ± 0.2	0.7 ± 0.3
Renal metabolism					
BUN (mg/dL)	"Yabukita"	12.8 ± 3.6	12.1 ± 2.8	12.5 ± 2.8	12.6 ± 3.0
	"Sunruge"	13.2 ± 2.6	11.7 ± 2.3	12.2 ± 2.5	12.3 ± 2.6
	Placebo	12.9 ± 2.8	12.0 ± 2.4	13.1 ± 3.0	12.8 ± 2.9
CRE (mg/dL)	"Yabukita"	0.8 ± 0.2	0.8 ± 0.2	0.8 ± 0.2	0.8 ± 0.2
	"Sunruge"	0.8 ± 0.2	0.8 ± 0.2	0.8 ± 0.2	0.8 ± 0.2
	Placebo	0.8 ±0.2	0.8 ± 0.1	0.8 ± 0.2	0.8 ± 0.1
UA (mg/dL)	"Yabukita"	5.5 ± 1.3	5.8 ± 1.4	5.7 ± 1.5	5.6 ± 1.5
	"Sunruge"	5.3 ± 1.5	5.3 ± 1.4	5.3 ± 1.3	5.3 ± 1.4
	Placebo	5.5 ± 1.4	5.4 ± 1.4	5.3 ± 1.2	5.2 ± 1.4
Iron metabolism					
UIBC (mg/dL)	"Yabukita"	251.2 ± 84.6	262.8 ± 78.7	255.1 ± 67.4	266.2 ± 73.0
	"Sunruge"	204.4 ± 72.5	231.6 ± 73.0	225.1 ± 75.4	236.3 ± 75.8
	Placebo	210.1 ± 57.8	240.1 ± 47.7	224.0 ± 61.3	226.7 ± 66.5
TIBC (mg/dL)	"Yabukita"	362.0 ± 57.9	373.0 ± 55.4	364.7 ± 53.6	369.6 ± 50.4
	"Sunruge"	335.5 ± 44.6	340.5 ± 45.6	331.1 ± 50.2	338.4 ± 50.8
	Placebo	332.2 ± 44.6	339.2 ± 41.1	329.2 ± 42.0	332.4 ± 43.3
Cytokines					
C-reactive protein (mg/dL)	"Yabukita"	0.11 ± 0.18	0.07 ± 0.10	0.07 ± 0.06	0.08 ± 0.08
	"Sunruge"	0.07 ± 0.12	0.08 ± 0.16	0.22 ± 0.94	0.11 ± 0.26
	Placebo	0.09 ± 0.16	0.09 ± 0.22	0.37 ± 1.30	0.09 ± 0.17
Creatine phosphokinase (U/L)	"Yabukita"	93.3 ± 31.7	105.6 ± 42.1	124.6 ± 71.5	108.3 ± 39.0
	"Sunruge"	99.0 ± 42.7	113.2 ± 70.4	96.4 ± 39.5	117.8 ± 76.1
	Placebo	114.7 ± 111.0	112.9 ± 81.5	114.0±81.8	101.8 ± 54.1
Serum-amylase (U/L)	"Yabukita"	128.3 ± 319.0	146.3 ± 408.2	137.9 ± 365.6	140.8 ± 378.6
	"Sunruge"	76.5±21.3	81.7 ± 21.9	77.2 ± 21.6	79.9 ± 21.8
	Placebo	72.4 ± 23.3	79.6 ± 30.8	73.1 ± 21.9	72.6 ± 21.0

Diet survey					
Total daily intake (kcal.day)	"Yabukita"	1,790.2 ± 482.5	1,758.6 ± 401.2	1,825.0 ± 531.1	1,767.0 ± 492.0
	"Sunruge"	1,846.4 ± 452.6	1,919.6 ± 499.3	1,905.9 ± 402.5	1,853.4 ± 410.3
	Placebo	1,776.0 ± 452.2	1,791.0 ± 462.3	1,822.3 ± 406.4	1,773.0 ± 333.6
Protein (g/day)	"Yabukita"	64.0 ± 20.5	64.0 ± 17.5	67.1 ± 21.6	63.4 ± 19.3
	"Sunruge"	65.9 ± 18.3	70.8 ± 22.3	67.7 ± 16.2	67.1 ± 17.0
	Placebo	63.5 ± 22.1	63.1 ± 18.5	65.4 ± 20.8	62.5 ± 16.5
Fat (g/day)	"Yabukita"	62.7 ± 21.6	62.0 ± 18.9	65.9 ± 25.9	63.8 ± 22.5
	"Sunruge"	66.4 ± 18.5	70.8 ± 21.9	67.5 ± 17.5	66.1 ± 18.8
	Placebo	61.0 ± 21.2	62.8 ± 19.5	62.7 ± 18.3	59.5 ± 14.7
Carbohydrate (g/day)	"Yabukita"	227.9 ± 59.6	223.8 ± 47.5	230.0 ± 60.6	223.8 ± 60.2
	"Sunruge"	231.1 ± 61.9	234.6 ± 63.8	238.2 ± 58.8	231.9 ± 60.1
	Placebo	232.1 ± 53.6	231.9 ± 61.2	236.5 ± 49.8	234.9 ± 46.2
Dietary fiber (g/day)	"Yabukita"	11.8 ± 4.0	11.4 ± 3.5	12.2 ± 3.9	12.1 ± 4.1
	"Sunruge"	12.1 ± 3.9	12.0 ± 4.1	12.1 ± 3.6	12.0 ± 3.8
	Placebo	13.3 ± 7.7	12.0 ± 3.9	12.4± 4.3	12.4 ± 4.6
Salt (g/day)	"Yabukita"	8.9 ± 3.7	8.7 ± 3.6	9.0 ± 3.6	8.9 ± 3.5
	"Sunruge"	8.7 ± 3.1	9.2 ± 3.3	9.1 ± 3.4	8.7 ± 2.9
	Placebo	8.7 ± 3.2	9.3 ± 3.2	9.3 ± 3.3	8.8 ± 2.6

WBC, white blood cell count; RBC, red blood cell count; Hb, hemoglobin; Ht, hematocrit; Plt, Platelet count;
AST, aspartate aminotransferase; ALT, alanine aminotransferase; ALP, alkaline phosphatase; LDH, lactate dehydrogenase;
γGPT, gamma-glutamic pyruvic transaminase; LAP, leucine aminopeptidase; ChE, cholinesterase; ZTT, zinc sulfate turbidity test
TP, total protein; A/G ratio, albumin/globulin ratio; T-Bil, total bilirubin; BUN,blood urea nitrogen; CRE, creatinine;
UA, uric acid; UIBC, unsaturated iron binding capacity ; TIBC, total iron binding capacity.
One-way ANOVA and the Tukey post-hoc test vs placebo, paired t-test vs 0 week was performed to analyse the values.
Statictically significant, *; p <0.05, **; p <0.01 vs placebo group, #; p <0.05, ##; p <0.01 vs 0 week

Table S3. Secondary outcomes after drinking "Sunrouge", "Yabukita", or barley tea

	Interventions	Week 0	Week 4	Week 8	Week 12
LDL-C (mg/dL)	"Yabukita"	130.1 ± 31.2	133.8 ± 31.4	127.7 ± 35.8	131.4 ± 32.0
	"Sunrouge"	132.3 ± 30.4	129.2 ± 32.8	124.9 ± 33.1 #	124.6 ± 28.8 #
	Placebo	135.8 ± 27.3	134.8 ± 23.9	128.7 ± 25.1 #	131.2 ± 33.1
Endothelial markers					
Adiponectin (mg/L)	"Yabukita"	11.1 ± 5.8	12.3 ± 7.4 ##	12.5 ± 6.7 ##	13.2 ± 7.7 ##
	"Sunrouge"	10.2 ± 4.8	10.6 ± 5.1 ##	10.5 ± 4.7	11.0 ± 5.1 ##
	Placebo	10.6 ± 5.0	11.1 ± 5.2	11.0 ± 5.0	11.2 ± 5.3 ##
High sensitivity CRP (mg/L)	"Yabukita"	0.06 ± 0.06	0.04 ± 0.05	0.06 ± 0.06	0.06 ± 0.06
	"Sunrouge"	0.02 ± 0.02	0.03 ± 0.03	0.05 ± 0.04	0.04 ± 0.04
	Placebo	0.04 ± 0.05	0.04 ± 0.03	0.04 ± 0.05	0.03 ± 0.03
ADMA (mmol/L)	"Yabukita"	0.7 ± 0.2	0.5 ± 0.1 ##	0.7 ± 0.3	0.7 ± 0.2
	"Sunrouge"	0.7 ± 0.1	0.5 ± 0.1 ##	0.7 ± 0.3	0.7 ± 0.2
	Placebo	0.7 ± 0.1	0.5 ± 0.1 ##	0.7 ± 0.2	0.6 ± 0.1
Homocysteine (mmol/L)	"Yabukita"	11.4 ± 3.4	-	-	12.1 ± 3.7 ##
	"Sunrouge"	10.9 ± 2.7	-	-	11.0 ± 2.7
	Placebo	11.6 ± 3.5	-	-	11.1 ± 3.7
Oxidation marker					
Oxidized LDL (U/L)	"Yabukita"	111.3 ± 44.2	130.6 ± 43.3 ##	107.2 ± 30.4	106.2 ± 41.4
	"Sunrouge"	117.0 ± 41.7	128.8 ± 53.3	104.0 ± 46.0 ##	107.8 ± 51.1 #
	Placebo	119.9 ± 43.0	124.5 ± 39.5	115.2 ± 36.9	111.5 ± 39.4
Urinary 8-OHdG (ng/mg CRE)	"Yabukita"	7.7 ± 3.4	10.0 ± 5.0 ##	10.2 ± 4.9 ##	10.9 ± 5.2 ##
	"Sunrouge"	7.4 ± 2.9	9.7 ± 3.8 ##	9.3 ± 3.3 #	10.1 ± 4.1 ##
	Placebo	7.7 ± 3.1	9.8 ± 4.8	9.1 ± 3.6 ##	10.6 ± 4.3 ##
Intraocular pressure right eye(mmHg)	"Yabukita"	14.3 ± 3.3	15.0 ± 3.3 #	16.3 ± 3.4 ##	16.3 ± 2.9 ##
	"Sunrouge"	14.0 ± 3.4	14.0 ± 2.9	15.7± 3.6 ##	15.7 ± 3.6
	Placebo	14.3 ± 2.2	14.0 ± 2.5	15.8 ± 2.5 ##	15.6 ± 3.0 ##
left eye(mmHg)	"Yabukita"	14.6 ± 3.1	15.1 ± 3.2	16.6 ± 3.3 ##	16.6 ± 3.4 ##
	"Sunrouge"	14.0 ± 3.4	14.4 ± 3.1 ##	15.2 ± 3.2 ##	15.9 ± 3.8
	Placebo	14.2 ± 2.3	14.1 ± 2.7	15.8 ± 3.2 ##	15.8 ± 3.4 ##
Peripheral blood flow					
Growth rate (%)	"Yabukita"	38.3 ± 7.7	42.7 ± 17.0	43.4 ± 9.4 ##	43.5± 13.2 #
	"Sunrouge"	41.7± 10.1	45.6 ± 13.5	41.9 ± 8.8	44.6 ± 10.5
	Placebo	41.5 ± 10.3	44.6 ± 15.7	41.2 ± 10.4	44.9 ± 11.0
Tine from the releasing of an avascularization to max blood flow (sec.)	"Yabukita"	24.7 ± 15.9	23.2 ± 17.7	28.1 ± 18.8	28.9 ± 21.4
	"Sunrouge"	20.4 ± 17.2	23.7 ± 16.4	25.7 ± 18.3	19.1 ± 21.4
	Placebo	24.7 ± 16.5	25.5 ± 17.0	31.1 ± 18.4	27.5 ± 21.2

Values are shown as the mean ± standard deviation.

LDL-C, low density lipoprotein cholesterol; CRP, C-reactive protein; ADMA, asymmetric dimethylarginine;
8-OHdG, -oxo-dG,8-hydroxy-2'- deoxyguanosine.

One-way ANOVA and the Tukey post-hoc test vs placebo, paired t-test vs 0 week was performed to analyze the values.

Statistically significant, #; p <0.05, ##; p <0.01 vs 0 week
